# Smooth or with a Snap! Biomechanics of Trap Reopening in the Venus Flytrap (*Dionaea muscipula*)

**DOI:** 10.1002/advs.202201362

**Published:** 2022-06-01

**Authors:** Grażyna M. Durak, Rebecca Thierer, Renate Sachse, Manfred Bischoff, Thomas Speck, Simon Poppinga

**Affiliations:** ^1^ Botanical Garden, Plant Biomechanics Group University of Freiburg Freiburg im Breisgau 79085 Germany; ^2^ Institute for Structural Mechanics University of Stuttgart Stuttgart 70550 Germany; ^3^ TUM School of Engineering and Design Department of Engineering Physics and Computation Technical University of Munich Garching b. München 85748 Germany; ^4^ Cluster of Excellence livMatS @ FIT – Freiburg Center for Interactive Materials and Bioinspired Technologies University of Freiburg Freiburg im Breisgau 79110 Germany; ^5^ Department of Biology Technical University of Darmstadt Botanical Garden Darmstadt 64287 Germany

**Keywords:** biomechanics, carnivorous plants, mechanical instability problems, plant movement, snap‐buckling, snap‐traps

## Abstract

Fast snapping in the carnivorous Venus flytrap (*Dionaea muscipula*) involves trap lobe bending and abrupt curvature inversion (snap‐buckling), but how do these traps reopen? Here, the trap reopening mechanics in two different *D. muscipula* clones, producing normal‐sized (N traps, max. ≈3 cm in length) and large traps (L traps, max. ≈4.5 cm in length) are investigated. Time‐lapse experiments reveal that both N and L traps can reopen by smooth and continuous outward lobe bending, but only L traps can undergo smooth bending followed by a much faster snap‐through of the lobes. Additionally, L traps can reopen asynchronously, with one of the lobes moving before the other. This study challenges the current consensus on trap reopening, which describes it as a slow, smooth process driven by hydraulics and cell growth and/or expansion. Based on the results gained via three‐dimensional digital image correlation (3D‐DIC), morphological and mechanical investigations, the differences in trap reopening are proposed to stem from a combination of size and slenderness of individual traps. This study elucidates trap reopening processes in the (in)famous *Dionaea* snap traps – unique shape‐shifting structures of great interest for plant biomechanics, functional morphology, and applications in biomimetics, i.e., soft robotics.

## Graphical abstract

The snap traps of the infamous Venus flytrap have remarkable shape‐shifting capabilities. After fast snapping in an attempt to capture prey, the traps can slowly reopen via two modes of sequential deformation. Whereas normal‐sized traps (N traps) reopen smoothly and continuously via hydraulic processes alone, large and slender traps (L traps) can incorporate an additional reverse snap‐buckling instability during reopening.

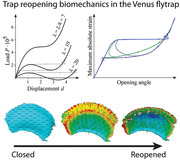

## 1 Introduction

The Venus flytrap (*D. muscipula*, Droseraceae) is a carnivorous plant native to subtropical wetlands on the east coast of North America.^[^ ^1^, ^2^, ^3^ ^]^ It gained notoriety due to its spectacular ability to capture prey via snap trap closure, which can be executed in as little as 100 ms.^[^ ^3^ ^]^ The fast snapping motion relies on a “smart” combination of hydraulic cellular processes with rapid release of stored elastic energy arising from prestress due to internal growth processes and hydrostatic pressure, which trigger the snap‐through.^[^ ^3^, ^4^, ^5^ ^]^ The snap‐through motion itself is a mechanical instability phenomenon affecting the doubly curved lobes of the traps.^[^ ^4^ ^]^ In contrast, trap reopening is a much slower process, of which duration depends on whether the trap remained empty following closure or if a food item was successfully trapped following mechanical stimulation by the prey (mainly arthropods). In the former scenario, the empty trap will reopen within 16–44 h in preparation for another hunting cycle.^[^ ^6^, ^7^ ^]^ In the latter scenario, the trap forms a digestive cavity to absorb nutrients from the prey, which extends the reopening time to 5–7 days on average.^[^ ^6^, ^8^ ^]^ In this study, we focused on the former scenario, mechanically triggering traps to snap but without offering any prey. Thus far, the particulars of the mechanism of trap reopening in *D. muscipula* remain speculative and center on the assumption that it is driven mainly by turgor and cellular growth.^[^ ^6^, ^7^, ^9^, ^10^ ^]^ Since trap size and geometry were found to strongly influence the fast snapping process, it is likely that these parameters influence trap reopening as well.^[^ ^3^ ^]^ We, therefore, elected to test this by employing *D. muscipula* clones which produced traps within two size classes: normal‐sized (N) and large‐sized (L). We thus set out to investigate the process further to provide more insight into deformation changes affecting trap surfaces, plant behavior, as well as overall plant morphometrics pertinent to the trap reopening process.

## 2 Results and Discussion

### 2.1 Trap Reopening: N Versus L Traps Producing Plant Clones

Traps produced by the N and L plants had a median length of 23 and 32.71 mm respectively, and the maximum size of N traps did not exceed 30 mm (**Table** **1**). The L plants produced significantly larger traps (*t* = −11.95, *p* < 0.001), exceeding 40 mm in maximum length (Table 1). Time‐lapse observations of trap reopening indicated that the process differs in duration as well as in lobe deformation between these two morphologically distinct clones. N traps opened exclusively via a smooth, homogenous outwards bending motion, which is consistent with previous reports in literature.^[^ ^6^, ^7^ ^]^ N traps required a median reopening time of 27.31 h. In contrast, the L traps required a median reopening time of 31.45 h, and either followed the smooth, homogenous bending reopening scenario observed in the N plants (Time‐lapse S1, Supporting Information), or underwent an initially slow and homogenous stage, followed by reverse snap‐buckling (Time‐lapse S2, Supporting Information). Longer reopening times for L plants are consistent with the theory that trap reopening process relies heavily on hydraulic actuation.^[^ ^10^, ^11^ ^]^ Assuming that the process relies on the least metabolically costly way of water transport – transpiration – it stands to reason that the large L traps with a lower surface‐to‐volume ratio than N traps would take longer to reopen than the smaller ones.^[^ ^12^ ^]^

**Table 1:** Size characterization of traps produced by the N and L plant clones. Trap lengths and heights were measured as specified in Figure 3A, D on *n* = 50 traps for each *D. muscipula* clone

Plant clone	Median reopening times, *n*_N_ = 35, *n*_L_ = 41, [h]	Median trap length *n* = 50 [mm]	Median trap height *n* = 50 [mm]	Min. trap length [mm]	Max. trap length [mm]
N	28.07	23	11.23	14.55	28.05
L	31.45	32.72	15.17	23.95	43.10

Additionally, a “rim popping” motion can occur right at the edge of the trap in both N and L plants during the initial reopening stage (Time‐lapse S3, Supporting Information). It is most likely due to friction of the interlocking cilia (“teeth”) sliding apart as well as the deposits of sticky nectar produced around the rim of the trap.^[^ ^13^, ^14^ ^]^ We observed that 3.85% of L traps (*n* = 41) reopen via reverse snap‐buckling, with the remainder of traps reopening in a smooth fashion analogous to all N traps. The reverse snap‐buckling process itself was significantly slower to that observed during rapid trap closure and took between 6–60 min, thus constituting a distinct stage during the slow reopening process in *D. muscipula*.

### 2.2 Strain Distribution

**Figure 1:**
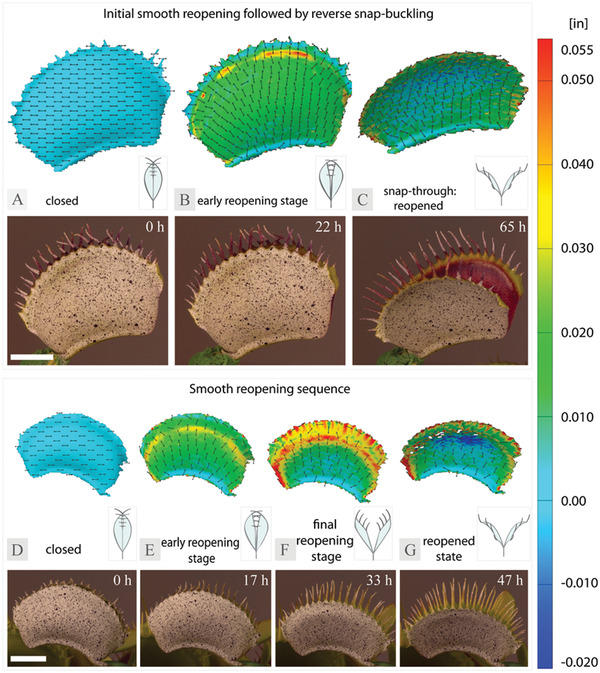
Major strain distribution and evolution in *x*‐direction, computed as true strain on a 3D surface reconstruction of the outer surfaces of L trap lobes throughout the reopening process (top) and a corresponding digital image of the plant (bottom) featuring smooth initial reopening followed by A–C) reverse snap‐buckling D–G) and smooth reopening only. Insets represent a schematic of a trap reopening stage in the cross‐section view. Scale bars correspond to 0.5 in.

**Figure 2:**
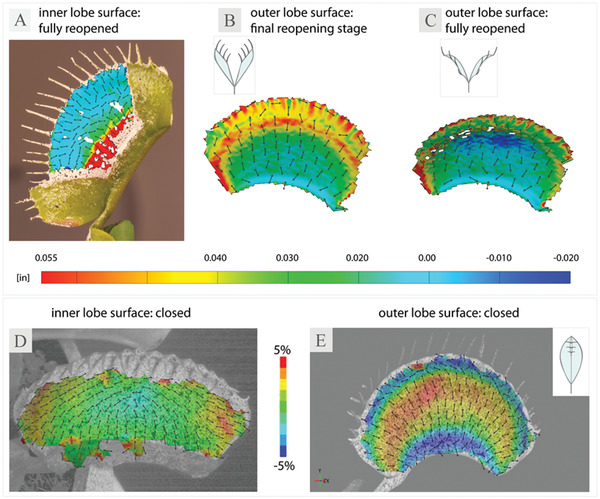
Comparison of major strain distribution in *x*‐direction during trap reopening and closure. Major strain was computed as true strain on a 3D surface reconstruction of B,C) the outer and A) inner trap lobe surfaces of L traps. Strain distribution during trap closure was calculated as technical strain on D) the inner and E) outer lobe surface of N trap lobes. Insets indicate trap reopening stage in cross‐section. Images D and E were adapted with permission from Sachse et al..^[^ ^4^ ^]^ Plain digital images from a corresponding time point for images A–C can be found in Figure S1A–C, Supporting Information.

Examination of the evolving strain patterns in *x*‐direction on the outer and inner surfaces of individual *D. muscipula* trap lobes indicate that both abaxial and adaxial tissue layers are involved in trap reopening and undergo localized shrinking and expansion in specific areas over time (**Figure** **1**, **Figure** **2**, smooth reopening: Time‐lapse S4, Supporting Information, reverse snap‐buckling: Time‐lapse S5, Supporting Information). L traps showed a consistent pattern of an increase in strain around the edges of the outer trap lobe surfaces, directed perpendicular to the midrib during both reopening scenarios (purely smooth, and smooth with a subsequent snap‐through) (Figure 1A,D–F). The area close to the midrib remained neutral during the final reopening stage, whereas the tissue above the trap midpoint continued to expand in a crescent pattern forming a distinct ring around the edges of the trap (Figure 1B,F). Following the final reopening step, tissue above the lobe midpoint experienced negative strain towards the rim of the lobe (Figure 1C,G). Similar, although less pronounced, strain patterns occur on the outer lobe surfaces of the N plants (Figure S1D–E, Supporting Information).

In contrast, following trap closure, the outer trap surface of L traps experiences negative strain at the rim of the trap as well as in the midrib area, whereas the middle of the trap is subject to expansion (Figure 2B,C,E).^[^ ^4^ ^]^ Examination of the inner lobe surface in the L traps revealed an expansion field at the base of the trap, extending towards the middle of the trap lobe over time, with a positive strain directed perpendicular to the midrib and the remainder of the lobe staying neutral (Figure 2A). This is in line with the reverse strain patterns observed during trap closure, in which case only the tissue around the edges of the inner side of the trap lobe experiences positive strain (Figure 2D).^[^ ^4^ ^]^ Although there is some overlap in the strain patterns between trap reopening and closure in terms of areas of epidermis involved in the process, the observed patterns further indicate that slow reopening is not a simple reversal of the deformations occurring during fast closure. This stands in line with previous findings in the literature, showing that the epidermis undergoes length changes in different trap regions at different times, and yet following a full trap closure/reopening cycle, no overall differences in mean cell length can be found.^[^ ^9^ ^]^

### 2.3 Trap Morphometrics and Slenderness

**Figure 3:**
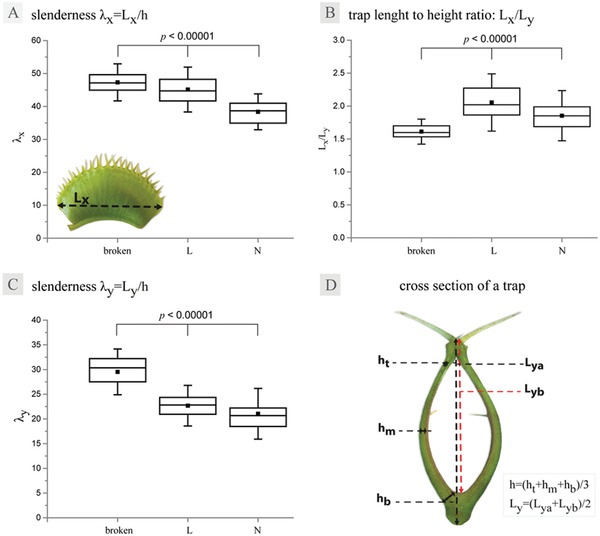
Box – whisker plots of the slenderness calculated for broken L traps, intact L traps and N traps: A) slenderness *λ_x_*, B) trap length to height ratio, C) slenderness *λ*_y_, whiskers represent standard deviation, *p*‐values refer to significant differences in given parameters between different trap types, confirmed with Kruskal–Wallis tests detailed in Table S2, Supporting Information. Schematic of measurements used in calculation of D) slenderness *λ*: *h*_b_ – lobe thickness at the bottom of the trap, *h*_m_ – lobe thickness in the middle of the trap, *h*_t_ – lobe thickness at the top of the trap.

To explain the differences between the trap reopening modes from a mechanical point of view, we investigated if trap morphology differed between the N and L plants in terms of the slenderness *λ*. In principle, slender structures are more likely to undergo snap‐through. For the purpose of this study we used a simplified calculation of trap lobe slenderness which we defined as analogous to an object with a flat surface, using lengths in both *x*‐ and *y*‐direction as well as thickness (**Figure** **3**A,D). Indeed, we found that slenderness in *x*‐direction was significantly different for traps of the N versus L plant clones (*t* = −8.22, *p* < 0.00001, *n* = 50; Table S1, Supporting Information), with median slenderness values of 38.67 and 44.74 for N and L morphotypes respectively. Given that the slenderness values have a high variation (Figure 3), it is possible that only traps with the highest slenderness undergo snap‐through during trap reopening, which would also contribute toward explaining why this phenomenon is rarely observed and has not been reported before.

Since the trap reopening mode involving reverse snap‐buckling is exclusive to large traps with comparatively high slenderness, we may speculate that previous investigations into trap reopening were presumably carried out on plants which did not fulfill these morphological criteria and instead produced normal‐sized or relatively small traps (cf. ref. [7] where all traps reopened smoothly). This is, however, difficult to determine, as – unfortunately – not all studies report the trap size of the plants used in experiments. Another factor, which could play an important role in whether the trap will or will not undergo snap‐through during reopening, could be related to the physiological status of the plant. Since *D. muscipula* is a subtropical species, its photosystem is adapted to high irradiance, at the same time it is sensitive to changes in light conditions.^[^ ^15^, ^16^ ^]^ During our cultivation of *D. muscipula* in the Freiburg Botanical Garden we observed that as the photoperiod shortens in autumn, the traps become sluggish and eventually cease to snap altogether. Since trap closure incurs relatively high energy costs if no prey is captured to compensate for the initial energy expenditure on the snapping motion, it can be expected that plants would follow the least energetically costly method of trap reopening.^[^ ^17^, ^18^ ^]^ It is possible that plants which could theoretically undergo snap‐through during trap reopening follow the more energetically efficient, purely transpiration‐driven smooth reopening scenario instead.

### 2.4 Mechanical Investigations on Slenderness and Equilibrium Paths of Instability Problems

**Figure 4:**
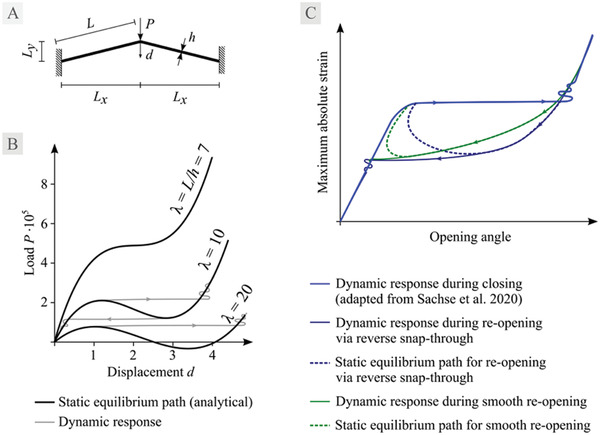
A) Shallow frame system as a mechanical prototype for snap‐through including bending deformations. B)Analytically derived static equilibrium paths for three different slenderness values and a sketch of the expected behavior of the dynamic snap‐through response. The snap‐through process for an unloading scenario is sketched for *λ* = 10. C) Two hypothetical equilibrium paths for closing and reopening of *D. muscipula*, based on dynamic response as simulated in Sachse et al.^[^ ^4^ ^]^ Both paths show identical closing behavior via snap‐through, whereas smooth motion or a reverse snap‐through can be observed during trap reopening.

The quantitative effect of a structure's slenderness on its instability behavior was previously reported, e.g., for a timber grid‐shell dome.^[^ ^19^ ^]^ On this basis, we devised and investigated a simplified mechanical model which – in contrast to former studies on a three‐hinged truss system by Sachse et al.^[^ ^4^ ^]^ – includes bending deformations similar to those occurring in *D. muscipula* trap lobes. **Figure** **4**A,B shows the mechanical setup for a shallow frame system and its load‐displacement characteristics, i.e., static equilibrium paths for three different values of slenderness  *λ*. The paths represent the exact analytical solution according to Bernoulli beam theory, valid for geometrically non‐linear static behavior with large rotations and small strains. Based on these static equilibrium paths, we can deduce the dynamic behavior during snap‐through. For lower slenderness values, i.e., more bulky structures, the path shows less pronounced gradient changes during the snap‐through phase up to an almost smooth behavior, for which the load value increases monotonously. Since every path shows symmetry, the dynamic behavior is alike during loading and unloading. This is in sharp contrast to the typical behavior in bifurcation problems including snap‐back, with a distinctly different behavior upon loading and unloading.

In a former finite element simulation of the snap‐through of *D. muscipula* traps, only the dynamic system response could be traced.^[^ ^4^ ^]^ Typical for instability problems, convergence issues impeded tracing of the complete equilibrium paths via non‐linear static analyses using the arc‐length method. Combining the dynamic response of *D. muscipula* traps with our findings regarding slenderness shown in Figure 4 B on different behavior during loading and unloading, we state two hypothetical equilibrium paths (Figure 4 C). For both paths, the deduced dynamic response during closing is shown as in Sachse et al.,^[^ ^4^ ^]^ whereas their dynamic response during reopening shows a different behavior, leading to either a smooth or reverse snap‐through process.

### 2.5 Trap Breakage

**Figure 5:**
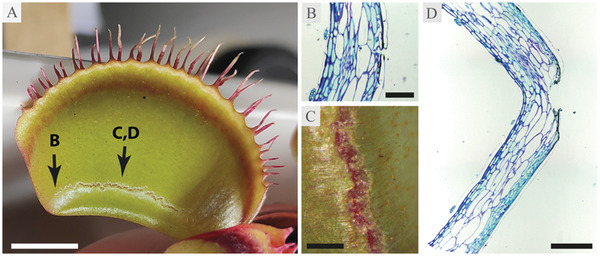
A) Photograph of an L trap following spontaneous breakage, B) Toluidine‐blue stained section of the tear taken at the edge, C) close‐up at the center of the tear, and D) Toluidine‐blue stained section in the middle of the tear. The trap was mechanically stimulated to snap, no prey was offered. Scale bars are as follows: A: 1 cm, B: 200 µm, C: 5 mm, and D: 500 µm.

We have observed traps of the L clone to occasionally break when reopening (Time‐lapse S6, Supporting Information). The breakage is consistently localized in the area close to the midrib, either in a semiparallel, crescent, or a diffuse pattern (**Figure** **5**A, Figure S2, Supporting Information). Although the outer lobe surface does not appear to experience much strain during reopening in the area where the tear forms (Figure 1), the strain appears on the inner surface of the lobe instead (Figure 2A), likely contributing a vulnerability to breakage in this area. The tear penetrates tissues deep into the mesophyll at the deepest point (Figure 5B–D), however, the inner lobe lining appears to remain intact. Further analysis of the broken traps revealed that they are not only significantly more slender in both *x*‐ and *y*‐direction than the intact L and N traps (Figure 3A,C; Table S1, Table S2, Supporting Information), but also exhibit a much lower trap length‐to‐height ratio (Figure 3B, Table S1, Supporting Information). The combination of higher slenderness as well as lower length‐to‐height ratio can also affect trap curvature. As, from a mechanical point of view, more curved and doubly curved structures can resist higher loads, this can be the cause for some of the traps tearing at the base during trap reopening. The observed breakage was only recorded in the more slender L trap type, which could indicate a potential limitation of trap functionality, dependent on the physical dimensions of the trap, thus imposing a quasi‐size limitation on the plant itself.

### 2.6 *D. muscipula* Snap‐Traps as a Biomimetic Model System

*D. muscipula* snap‐traps perform a variety of different motion sequences, ranging from hydraulically actuated fast curvature inversion from concave to convex following fast closure, through reopening to the initial “ready to snap” configuration, to the formation of a specialized digestive cavity, and then reopening yet again following digestion to complete the hunting cycle.^[^ ^3^, ^4^, ^9^ ^]^ Thus, the trap lobes perform the following geometrical transitions: concave to convex (fast snapping, duration in the millisecond regime), convex to concave (reopening from idle closed configuration, duration: hours regime), convex to flattened (stomach formation, duration: hours to days), and flattened to concave (reopening after prey digestion, duration: several days). The highly versatile shape‐shifting capability of *D. muscipula* snap‐traps is an inspiration for a number of artificial Venus flytrap systems, as well as soft‐robotics based demonstrators, grippers, optical devices relying on the bi‐stable snap‐buckling principle and programmable self‐shaping materials among other applications.^[^ ^20^, ^21^, ^22^, ^23^, ^24^ ^]^ Since the majority of the current research focuses on the fast snapping movement, our study on the much slower reopening process provides essential information toward further development of the aforementioned systems in terms of fast versus slow motion potential of the same structure. Therefore, our data not only contribute to the current Venus flytrap model systems, but is also of high relevance to compliant mechanisms and concept generators for engineering sciences.

## 3 Conclusion

Trap reopening mechanics of the *D. muscipula* snap‐traps do not simply follow trap closure stages in a reverse order, and are unlikely to be solely turgor/cell growth‐driven, as it was previously assumed. We show that the reopening scenario occurring in slender traps of L plants, where a smooth initial opening is followed by a relatively fast snap‐through is analogous to that occurring during fast trap closure and as such is at least partially actuated by the release of accumulated prestress. The duality of trap reopening behavior observed in the L plants stands in contrast to the current consensus in literature, in which trap reopening is viewed as a slow and relatively homogenous process. Instead, trap reopening appears to be mainly dependent on morphology of individual traps, i.e., their size, length‐to‐height ratio as well as slenderness. Reopening is additionally influenced by biological and physiological factors, contributing to changes in the observed behavior. Since only a small portion of the L specimens exhibit the ability to snap‐through during reopening, and the DIC measurements indicated similar strain distribution on the outer lobes of the traps which opened smoothly, the difference in the slenderness alone cannot sufficiently explain the reported trap behavior. Therefore, the existence of additional mechanical and physiological factors obstructing reverse snap‐buckling must also be considered in future investigations.

## 4 Experimental Section

### Plant Characterization and Culture Conditions

Two morphologically distinct clones of *D. muscipula*, determined based on trap size and behavior were selected for the purpose of this study. The clones, designated as N and L plants and traps were deposited at the University of Freiburg Herbarium under accession numbers FB15011 and FB15012 respectively. While the N plants were already cultivated and propagated asexually at the Freiburg Botanical Garden, the L plants were additionally purchased from Gartenbau Thomas Carow (Nüdlingen, Germany). All plants were maintained at the Freiburg Botanical Garden under recommended greenhouse conditions. Additionally, in order to ensure optimum growth, plants were fed with a paste prepared from lyophilized mealworms, coarsely smashed with a mortar and pestle, and reconstituted with distilled water.

### Statistical Analysis

Data distribution was checked for normality with a Shapiro–Wilk normality test. Normally distributed data was tested with a two‐sample *t*‐test with Welch correction. Not normally distributed data was tested with a two‐tailed Mann–Whitney *U*‐test. Kruskal–Wallis test was applied to datasets with uneven sample size and data distribution deviating from normal. Statistical analysis was carried out using OriginLab (OriginLab Corp., Northampton, USA) and GraphPad Prism (GraphPad Software Inc., San Diego, USA).

### Time‐Lapse Cinematography

For the duration of experiments, plants were moved from the greenhouse to a constant temperature chamber at 24.5 ± 1 °C with 40%–50% relative humidity and maintained under constant illumination with a custom‐made light source fitted with Lumilux L36 W/840 cool light tubes (Osram, Munich, Germany). Light levels were maintained between 90–120 µmol m^–2^ s^–1^ PPFD and monitored with a Li‐250 light meter (Li‐Cor Biosciences, Lincoln, USA). Plants were maintained in a tray with a minimum of 3 cm of distilled water. Time‐lapse image sequences were recorded with a USB camera (Conrad Electronics SE) at 2 min intervals and assembled into videos at 50 fps playback speed using FIJI/ImageJ (1.53f51) software.^[^ ^25^ ^]^ Video time‐lapses were then analyzed with FIJI/ImageJ, and times corresponding to the initial opening movement and the total duration of trap reopening measured until the lobe movement ceased were obtained. A total of *n* = 78 for large L traps and *n* = 80 for small N traps were screened during trap reopening process.

### Trap Morphometrics and Calculation of Trap Slenderness

Photographs of individual traps for the slenderness measurements were taken using a Fujifilm XT‐20 camera equipped with a XC 16–50 mm F3.5–5.6 OIS II lens (Fujifilm, Tokyo, Japan). Cross‐sections were cut with a razorblade and imaged using an Olympus SZX9 stereoscope equipped with a ColorView II camera (Olympus Corp., Tokyo, Japan). Lobe thickness *h* was calculated as an average of *h*_b_, measured at the base of the trap, at a right angle to the lobe, *h*_m_, measured at ½ *L*_ya_, and *h*_t_ measured at the top of the trap, right before the thickening where the cilia are located. *L*_ya_ was measured from the outer side of the trap midrib to the base of the cilia and *L*_yb_ was measured from the inner side of the trap bottom to the base of the cilia were averaged for each trap in order to get a more realistic value for trap height *L*_y_ in cross‐section. A detailed schematic of where the measurements were taken can be found in Figure 3D. *L*_x_ was measured from edge to edge of the lobe, at the widest point of the trap (Figure 3A inset).

### Trap Breakage Experiments

Broken traps were collected from 20 L plants maintained in the greenhouse. Every single trap on all 20 plants was mechanically triggered to snap without offering prey. All traps were screened following a 3‐day reopening cycle and all broken traps were collected for analysis as specified in the “Trap morphometrics and calculation of trap slenderness” section. The procedure was repeated 3 times and yielded a total of 31 traps.

### 3D Image Correlation (3D‐DIC)‐Based Surface Strain and Deformation Analysis

Time lapses of a single trap were recorded at 2 min intervals using PL‐D685CU cameras with 4.8 µm pixel size (Edmund Optics Inc. Barrington, New Jersey, USA), equipped with Makro–Planar *T* × 2 100 mm^−1^ ZF objective lenses (Carl Zeiss AG, Oberkochen, Germany). The cameras were set up at a stereo angle of 25.55° and a distance of 58 cm from the specimen placed on a custom‐made mount and synchronized via the Capture OEM software (v.2.3.7.9) by pixeLink (Rochester, New York, USA). The same software was used for image capture at 2 min intervals. Calibration was performed with a CQ2—30 × 24 GOM calibration panel, according to the protocol established for *D. muscipula* snap trap analysis.^[^ ^4^ ^]^ In order to enable tracking of individual points on the trap surface, the lobe was prepared by spraying with an antiglare Helling Laser Scanning Spray (Helling GmbH, Schleswig‐Holstein, Germany) and a stochastic speckle pattern was applied with a synthetic bristle brush and Mars Black acrylic paint (Winsor & Newton, London, UK). Individual traps were affixed to a wooden stick with plasticine, in order to prevent the reopening trap from moving out of camera view. The potted specimen was placed in a container with min. 3 cm of distilled water and illuminated with ≈120 µmol m^–2^ s^–1^ PPFD from a custom‐made light source fitted with Dulux L 80W/840 lamps (Osram, Munich, Germany). Traps were triggered to snap with a toothpick immediately before image acquisition. In order to perform measurements on the inner side of the lobe, a window in the middle of the trap was cut out using a sterile scalpel and immediately sealed with Vaseline. The plant was then allowed to reopen the cut trap and recover in the greenhouse for a minimum of 3 days. After the recovery period, the trap was prepared for surface analysis with DIC following the protocol analogous to the outer surface preparation. Only traps showing typical behavior to intact plants were analyzed. The data was then processed with Aramis Professional software (GOM GmbH, Braunschweig, Germany) for surface deformation analysis, using 24 × 17 and 18 × 14 pixel facets. Subsequently, major strain, calculated as true strain and major strain direction were analyzed on the outer and inner surfaces of the trap lobes. All experiments were carried out in a controlled temperature and humidity chamber at 24.5 ± 1 °C and between 40%–50% relative humidity.

### FAA Fixation and Sectioning of Broken Traps

Sections of 2–3 mm thickness were cut from both sides of the initial tear points as well as from the middle point of the tear, using a sterile scalpel. Sections were then treated with 60% FAA solution for 4 h. Subsequent sample preparation followed a standard FAA protocol. Samples were embedded in Technovit 7100 resin (Kulzer GmbH, Hanau, Germany) and then cut into 100 µm sections on a SLEE CUT 5062 microtome (SLEE medical GmbH, Mainz, Germany), stained with Toluidine‐blue and imaged on an Olympus BX61 light microscope (Olympus Corp., Tokyo, Japan).

### Derivation of Static Equilibrium Paths for a Shallow Frame System

Assuming small strains, a linear elastic material with Young’s modulus *E* is chosen for the beams of the system as shown in Figure 4 A. The cross‐sectional area *A*  =  *bh* and the second moment of area $I=\frac{bh^{3}}{12}$ result in an axial stiffness of *EA* and a bending stiffness of *EI* for the beams. The engineering strain is defined as

(1) $$\varepsilon =\frac{l-L}{L}$$

relating the length of a beam in the deformed configuration *l* to its initial length *L*. For the deformed configuration, *l* is obtained from the Pythagorean theorem as

(2) $$l=\sqrt{{L_{x}}^{2}+{\left(L_{y}-d\right)}^{2}}$$

According to the concept of a geometrically non‐linear analysis, the equilibrium of forces in the deformed configuration is used at the top of the frame:

(3) 2Nsinφ−2Vcosφ+P=0

with $\tan\varphi =\frac{L_{y}-d}{L_{x}}$, normal force *N*  =  *εEA* and transverse shear force $V=\frac{12\mathrm{EI}}{l^{3}}\cos\varphi d$. Combining the equations and solving for the external force yields a non‐linear relationship between external force *P* and displacement *d*, i.e., a static equilibrium path:

(4) $$\begin{array}{ccc} P & = & \frac{2\mathrm{Eb}h^{3}dL_{x}^{2}\sqrt{L_{x}^{2}+{\left(-L_{y}+d\right)}^{2}}}{\sqrt{L_{x}^{2}+{\left(-L_{y}+d\right)}^{2}}L^{3}\left(L_{x}^{2}+{\left(L_{y}-d\right)}^{2}\right)}\\ & & -\;\frac{2\mathrm{Ebh}L^{2}\left(-L_{y}+d\right)\left(L_{x}^{2}+{\left(L_{y}-d\right)}^{2}\right)\left(-\sqrt{L_{x}^{2}+{\left(L_{y}-d\right)}^{2}}+L\right)}{\sqrt{L_{x}^{2}+{\left(-L_{y}+d\right)}^{2}}L^{3}\left(L_{x}^{2}+{\left(L_{y}-d\right)}^{2}\right)} \end{array}$$

Young's modulus *E* does not change the characteristics of the path, but merely enters as a linear scaling factor, as demonstrated above. To examine the influence of a structure's slenderness $\lambda =\frac{L}{h}$ on the path's characteristics, as shown in the three example paths in Figure 4B, *L_x_* =  10,   *L_y_* =  2 for the lengths, *b*  =  0.2 for the width of the beams and *E*  =  2.1 · 10^8^ is chosen, while adjusting the height *h* of the beams so that three slenderness values *λ*  = {7, 10, 20}  are obtained.

## Conflict of Interest

The authors declare no conflict of interest.

## Supporting information

Supporting Information

Supplemental Video 1

Supplemental Video 2

Supplemental Video 3

Supplemental Video 4

Supplemental Video 5

Supplemental Video 6

## Data Availability

The data that support the findings of this study are available from the corresponding author upon reasonable request.
