# Supplementary material for: Smooth or with a Snap! Biomechanics of Trap Reopening in the Venus Flytrap (Dionaea muscipula)
Source: Adv Sci (Weinh). 2022 Jun 1;9(22):2201362. doi: 10.1002/advs.202201362 (PMC9353449; doi:10.1002/advs.202201362)
Supplement: Supplementary file 1 — Supporting Information [file ADVS-9-2201362-s004.pdf]

Supporting Information

Title

Smooth or with a snap! Biomechanics of trap reopening in the Venus flytrap (*Dionaea muscipula*)

Grażyna M. Durak, Rebecca Thierer, Renate Sachse, Manfred Bischoff, Thomas Speck and Simon Poppinga*

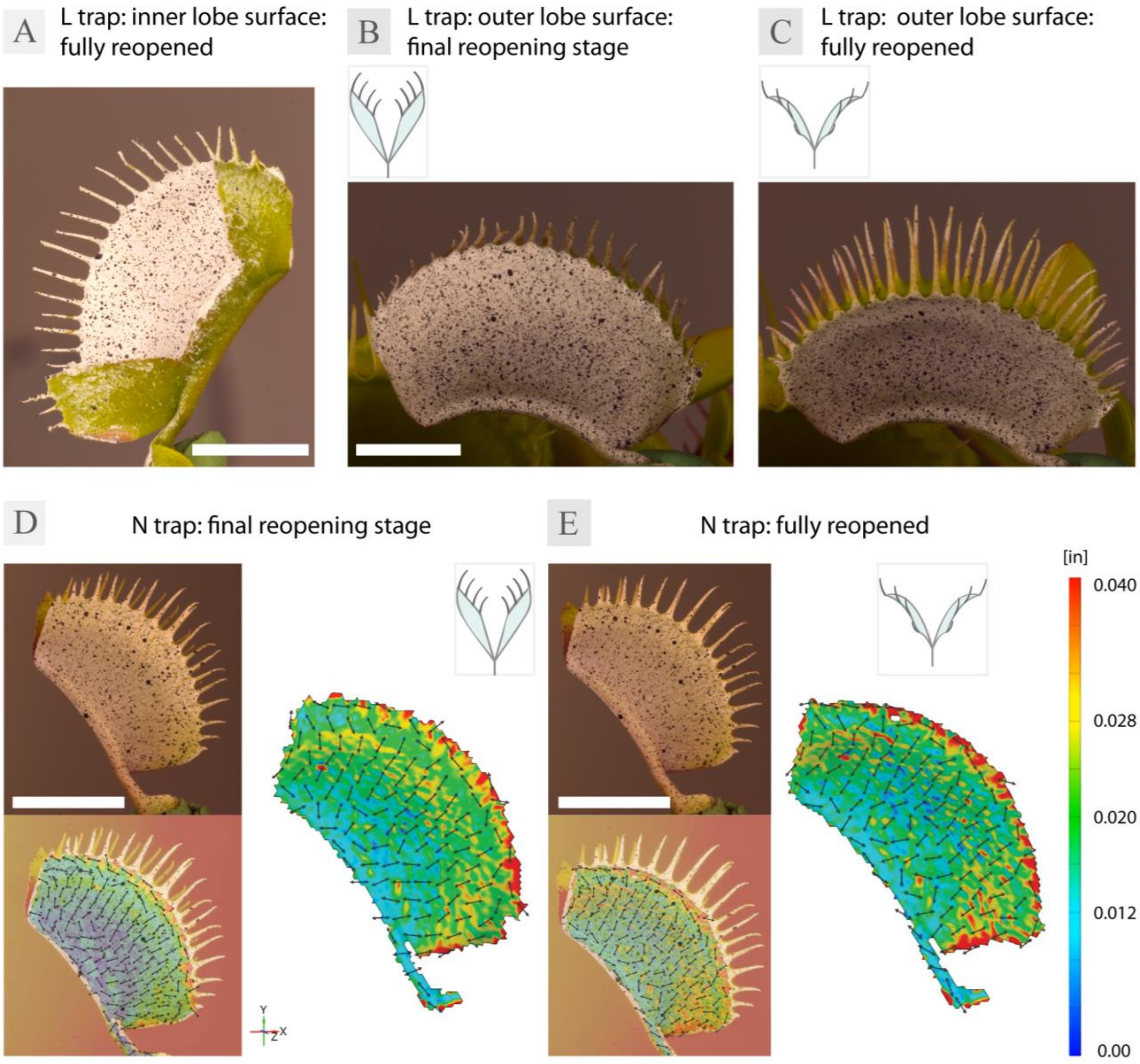

Figure S1. Digital images of the L traps from **Figure 2A-C** corresponding to their respective surface projections (A-C) and N traps showing a typical major strain distribution in x-direction, computed as true strain on a 3D surface reconstruction of the outer surface of the trap lobes throughout trap reopening cycle (D,E). Insets in D,E depict an actual digital image of the reopening trap (above), an overlay of major strain distribution from the corresponding time point in the time-lapse (below) as well as a sketch of trap reopening stage in cross-section view (upper right corner). Scale bars corresponds to 0.5 inch.

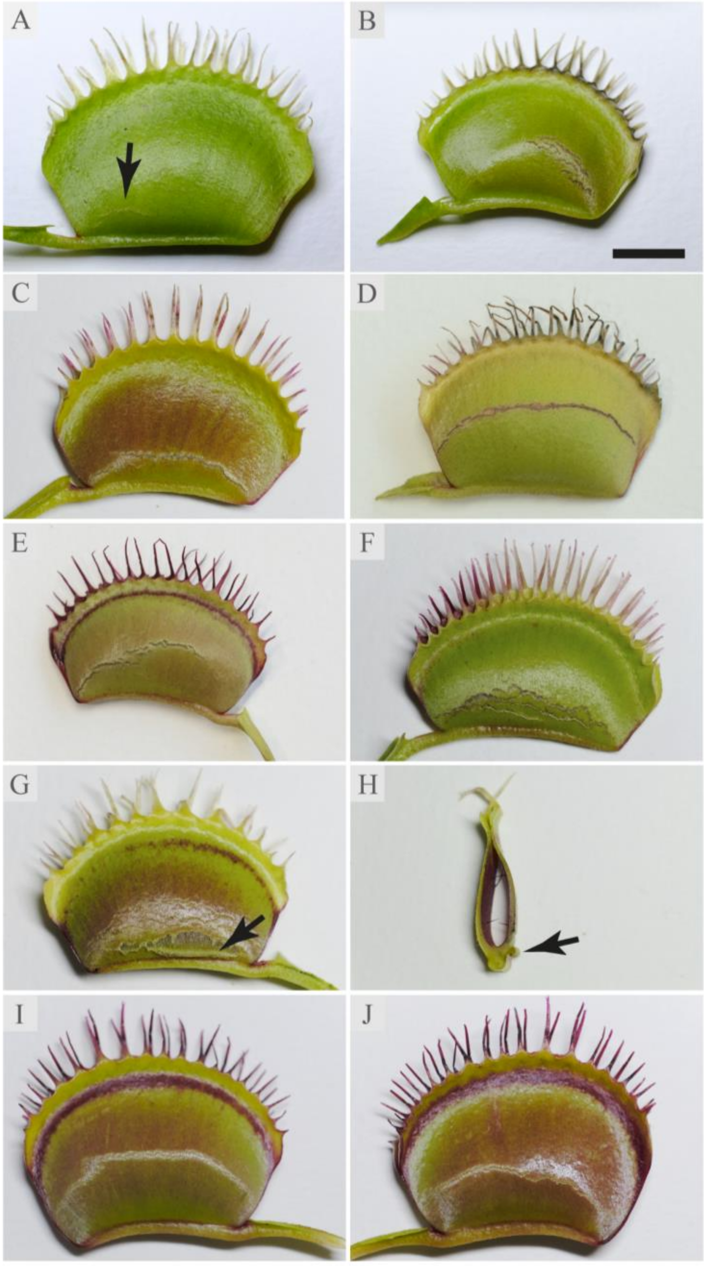

Figure S2. Different patterns of trap breakage in the L morphotype of *D. muscipula*. A, B – partial tear at one side of the trap lobe (arrow); C – crescent tear ending at the trap edges close to the midrib; D – a tear located just above the middle of the trap in L_y direction, tapering down at the trap edges; E – semi-crescent tear, F – multiple tears forming a crescent pattern located close to the midrib; G – multiple tears parallel to the midrib with epidermis peeling off due to the tear (arrow), H – cross section of the trap from image G, with the torn epidermis curled downwards (arrow); I,J – both trap lobes torn in a crescent pattern, just below the middle of the trap lobe. Scale bar = 1 cm.

The incidence of broken traps

Due to the very large number of traps screened during the experiment (10-20 traps per plant giving an average of 300 traps/replicate when using 20 plants; 900 traps in total), the exact number of traps was not individually counted and instead an approximation is provided. Given that each one of the three screening cycles yielded between 9-11 broken traps (a total of 31) we estimate that an average of 3.44% of traps break during the reopening cycle.

Table S1. Values of slenderness λ_x and λ_y, trap length L_x and height L_y and average thickness h. Values provided for the broken L traps, as well as L and N plant morphotypes; n_N and n_L = 50, $n_{L,broken}$ = 31 individual traps.

	**Mean N**	**Mean L**	**Mean broken**	**Median N**	**Median L**	**Median broken**
**Slenderness λ_x**	38.37	45.13	47.32	38.67	44.74	47.14
**Slenderness λ_y**	21.05	22.69	29.52	20.67	22.83	30.35
**Length L_x**	22.89	32.91	35.83	23.00	32.72	36.64
**Height L_y**	12.55	16.51	22.37	12.72	16.17	22.37
**L_x/L_y**	1.85	2.05	1.61	1.85	2.02	1.60
**h**	0.60	0.73	0.76	0.61	0.73	0.79

Table S2. Kruskal-Wallis test results. Dataset for N and L traps: n = 50, dataset for broken L traps: n = 31.

	**H**	***p***	**N**
**Slenderness λ_x**	66.3629	< 0.00001	131
**Slenderness λ_y**	63.2132	< 0.00001	131
**L_x/L_y**	49.7561	< 0.00001	131

List of time-lapses

Frame rate: 2 min

Time-Lapse S1: smooth trap reopening, playback speed 50 fps, scale bar = 1 cm

Time-Lapse S2: trap reopening with reverse snap-buckling, playback speed 50 fps, scale bar = 1 cm

Time-Lapse S3: trap reopening with initial “rim-popping”, playback speed 50 fps

Time-Lapse S4: surface projection of a trap and evolution of strain in x-direction during smooth trap reopening, playback speed 215 fps

Time-Lapse S5: surface projection of a trap and evolution of strain in x-direction during trap reopening featuring reverse snap-buckling, playback speed 215 fps

Time-lapse S6: trap breakage during attempted trap reopening, playback speed 50 fps
